# Glycogen accumulation in adipocyte precursors from elderly and obese subjects triggers inflammation via SIRT1/6 signaling

**DOI:** 10.1111/acel.13667

**Published:** 2022-07-10

**Authors:** Margarida Terrón‐Puig, Isabel Huber‐Ruano, Joan Sabadell‐Basallote, Miriam Ejarque, Catalina Núñez‐Roa, Elsa Maymó‐Masip, Rosa Jorba, Carolina Serena, Joan Vendrell, Sonia Fernández‐Veledo

**Affiliations:** ^1^ Institut d'Investigació Sanitària Pere Virgili (IISPV), Endocrinology and Nutrition Service Hospital Universitari de Tarragona Joan XXIII Tarragona Spain; ^2^ CIBER de Diabetes y Enfermedades Metabólicas Asociadas (CIBERDEM)‐Instituto de Salud Carlos III (ISCIII) Madrid Spain; ^3^ Rovira i Virgili University Tarragona Spain; ^4^ Servei d'Anàlisis clíniques. Laboratori Clínic ICS Camp de Tarragona‐Terres de l'Ebre Hospital Universitari de Tarragona Joan XXIII Tarragona Spain; ^5^ Servei de Cirugia General i de l'Aparell Digestiu, Hospital Universitari de Tarragona Joan XXIII Institut d'Investigació Sanitària Pere Virgili (IISPV) Tarragona Spain

**Keywords:** adipose‐derived mesenchymal stromal cells, Aging, glycogen, glycolysis, inflammation, obesity, SIRT1, SIRT6

## Abstract

Dysfunctional adipocyte precursors have emerged as key determinants for obesity‐ and aging‐related inflammation, but the mechanistic basis remains poorly understood. Here, we explored the dysfunctional adipose tissue of elderly and obese individuals focusing on the metabolic and inflammatory state of human adipose‐derived mesenchymal stromal cells (hASCs), and on sirtuins, which link metabolism and inflammation. Both obesity and aging impaired the differentiation potential of hASCs but had a different impact on their proliferative capacity. hASCs from elderly individuals (≥65 years) showed an upregulation of glycolysis‐related genes, which was accompanied by increased lactate secretion and glycogen storage, a phenotype that was exaggerated by obesity. Multiplex protein profiling revealed that the metabolic switch to glycogenesis was associated with a pro‐inflammatory secretome concomitant with a decrease in the protein expression of SIRT1 and SIRT6. siRNA‐mediated knockdown of *SIRT1* and *SIRT6* in hASCs from lean adults increased the expression of pro‐inflammatory and glycolysis‐related markers, and enforced glycogen deposition by overexpression of protein targeting to glycogen (PTG) led to a downregulation of SIRT1/6 protein levels, mimicking the inflammatory state of hASCs from elderly subjects. Overall, our data point to a glycogen‐SIRT1/6 signaling axis as a driver of age‐related inflammation in adipocyte precursors.

## Graphical abstract

Glycogen deposition in adipocyte precursors as a driver of inflammation through a SIRT1/6‐dependent mechanism. Both aging and obesity impact the metabolic and inflammatory properties of adipocyte precursors. A novel pathway linking metabolic dysregulation and inflammation is shown.

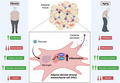

## 1 INTRODUCTION

An increase in life expectancy and an aging population, together with the unabated epidemic of obesity, represent one of the greatest health challenges facing our society. Obesity and aging are interrelated and influence each other, which worsens the economic and social burden of these conditions. The risk of obesity increases with age and, conversely, obesity can accelerate aging and increase the risk of early mortality **(**Tzanetakou et al., 2012). It is now appreciated that aging (a physiological state) and obesity (a pathological state) share similar biological hallmarks including metabolic dysregulation, weakened immunity, and systemic inflammation, which are all pathological phenotypes that occur with dysfunctional adipose tissue (AT) (Pérez et al., 2016).

AT is considered one of the largest and more plastic organs, with important immune and endocrine functions beyond energy storage (Stolarczyk, 2017). Obesity and aging are known to disturb AT metabolism as a consequence of AT expansion or senescence, respectively, leading to local inflammation and, ultimately, to a systemic and chronic state of inflammation (Mancuso & Bouchard, 2019). The mechanistic basis of these disturbances remains, however, unclear. Enhanced glycolytic flux and glycogen deposition appear to be overlapping metabolic abnormalities under certain aging (Wiley & Campisi, 2016) (Seo et al., 2008) and obesity (Serena et al., 2016) (Ceperuelo‐Mallafré et al., 2016) contexts. Along this line, we recently reported a causal relationship between glycogen deposition in adipocytes and inflammation (Ceperuelo‐Mallafré et al., 2016).

Within AT, human adipose‐derived mesenchymal stromal cells (hASCs) function as precursors of differentiated adipocytes and also have important immunoregulatory properties that are crucial for tissue homeostasis (Maria Spaggiari & Moretta, 2013). Both obesity (Pachón‐Peña et al., 2016) and aging (Turinetto et al., 2016) compromise the adipogenic potential of hASCs and modify their immune function (Zhang et al., 2021) (Serena et al., 2016). Interestingly, there is increasing evidence that hASCs are predominantly responsible for the changes in the secretory profile of AT that are induced by obesity (Siklova‐Vitkova et al., 2012) (Chung et al., 2006). This raises the question that the secretion of cytokines and chemokines in the context of aging or obesity might induce an inflammatory response in neighboring adipocytes, impairing lipid handling (Chung et al., 2006).

First discovered as NAD+‐dependent epigenetic regulators in yeast, sirtuins have emerged as bioenergetic sensors at the crossroads of metabolism and inflammation and are considered as crucial gatekeepers of tissue homeostasis during stress responses (Vachharajani et al., 2016). Failure of these systems to recover can lead to chronic inflammatory diseases. Of note, both obesity (Song et al., 2013) and aging (Khanh et al., 2018) have been associated with a fall in SIRT1 activity in AT, which in turn has been related to aberrant inflammation (Gillum et al., 2011).

In the present study, we sought to investigate the dysfunctional AT in relation to age and obesity in adults, focusing on the metabolic and inflammatory status of hASCs as key drivers of AT homeostasis. We report a new signaling pathway through which changes in the metabolic profile of hASCs as a consequence of obesity and aging regulate inflammation. Specifically, we found that hASCs from elderly and obese subjects exhibit an aberrant glycolytic flux concomitant with enhanced conversion of glucose to glycogen, which drives a pro‐inflammatory phenotype via a SIRT1/6‐dependent mechanism.

## 2 RESULTS

### 2.1 Obesity and aging differentially impact the proliferative but not the differentiation capacity of hASCs

Obesity is known to influence hASC plasticity (Pérez et al., 2016); however, whether aging also has an impact is less clear. We first examined the growth and proliferation of hASCs from donors stratified by age and body weight index (BMI) into the following groups: adult (>20 and < 65 years) or elderly (≥65 years), and lean (BMI < 25 kg/m^2^) or obese (BMI≥30 kg/m^2^), and the isolated hASCs were divided into the following four groups on this basis: lean adult (LA), lean elderly (LE), obese adult (OA), and obese elderly (OE).

**FIGURE 1:**
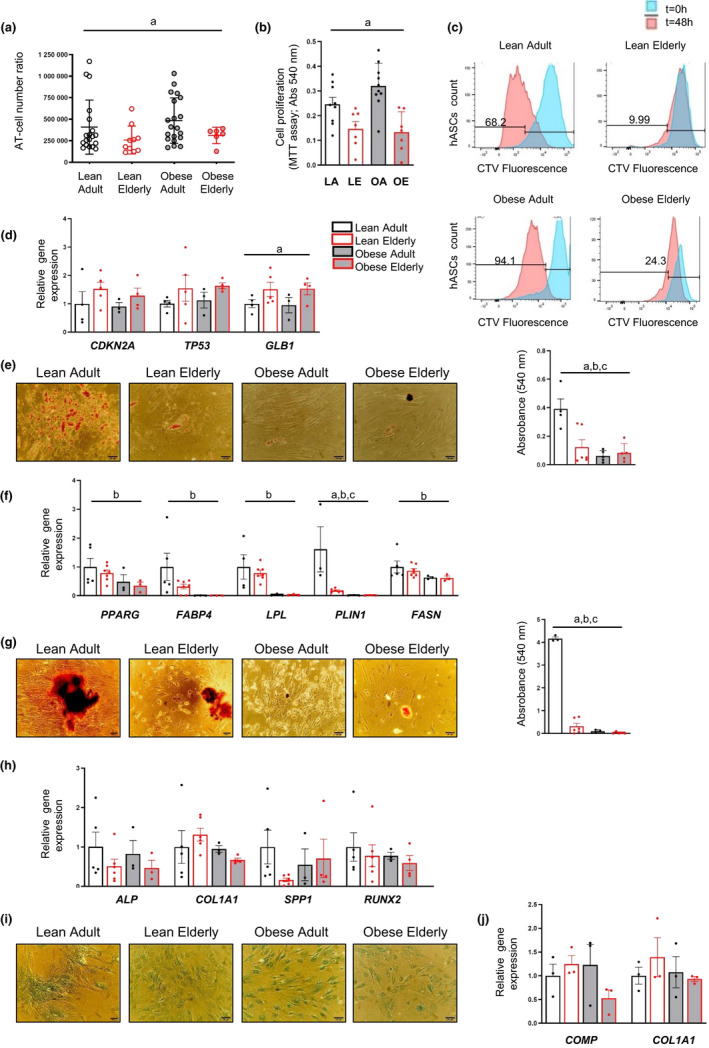
hASCs modified by aging or obesity show differences in proliferation and differentiation capacity. (a) Adipose tissue (AT)‐cell number ratio in all groups (*n* = 6–20). (b,c) Proliferation measured by MTT reduction after 7 days in culture (b) (*n* = 7–10) and CTV dye dilution after 48 h of incubation (c) (*n* = 1). (d) Gene expression of the senescence markers *CDKN2A*, *TP53,* and *GLB1* in hASCs (*n* = 3–5). (e,f) Representative images and quantification of Oil Red O staining (e) (*n* = 4–6) and relative gene expression of adipogenic (*PPARG, FABP4, LPL, PLIN1,* and *FASN*) markers (f) (*n* = 3–7) in adipocytes differentiated from hASCs. (g,h) Representative images and quantification of Alizarin Red staining (g) (*n* = 3–6) and relative gene expression of osteogenic (*ALP, COL1A1, SPP1,* and *RUNX2*) markers (h) (*n* = 3–6) in osteocytes differentiated from hASCs. (i,j) Representative images of Alcian Blue 8GX staining (i) and relative gene expression of chondrogenic (*COMP* and *COL1A1*) markers (j) (*n* = 3) in chondrocytes differentiated from hASCs. All images were taken at ×200 magnification; scale bar 100 μm. Data are shown as mean ± SEM; results of two‐way ANOVA were age *p* < 0.05 (a), BMI *p* < 0.05 (b), interaction between age and BMI *p* < 0.05 (c). Abbreviations: LA, lean adult; LE, lean elderly; OA, obese adult; OE, obese elderly

In accordance with our previous results demonstrating that obese‐derived hASCs have a higher proliferation rate than their lean‐derived counterparts (Pachón‐Peña et al., 2016), we found that the AT‐cell number ratio (number of proliferating hASCs at passage [P]0 per gram of digested AT) was significantly higher in the OA group than in the LE group (Figure 1a). By contrast, a lower AT‐cell number ratio was found in the groups of elderly subjects compared with the LA group independently of obesity (Figure 1a). Analysis of proliferation assessed by MTT reduction (Figure 1b) and by flow cytometric analysis of intracellular Cell Trace Violet (CTV) dilution (Figure 1c) confirmed the negative effect of aging on hASC proliferation, even in a background of obesity, suggesting that aging has a predominant effect over obesity for proliferation. We employed a general linear model to question whether proliferation (measured by MTT reduction) was significantly different between groups after adjusting for sex. This analysis revealed significant differences for MTT values between the four groups (*p* < 0.001) independent of sex (*p* = 0.490). We also used multiple linear regression analysis (stepwise forward selection procedures) to evaluate the potential role of age, sex, and BMI as independent factors associated with proliferation (MTT assay). Notably, we found that age was the main determinant of MTT values (R2 = 0.304; ß = −0.552, *p* < 0.001). Finally, examination of senescence‐related protein markers in cell extracts revealed an overall trend for greater expression in hASCs from elderly subjects (Figure 1d), which was significant for GLB1 expression.

We next analyzed the influence of aging on the multilineage differentiation potential of hASCs. Cells isolated from elderly subjects with or without obesity were significantly impaired in their capacity to differentiate into adipocytes, as revealed by Oil Red O staining of neutral lipids (Figure 1e) and by the gene expression of common adipogenic markers (*PPARG, FABP4, LPL, PLIN1,* and *FASN*) (Figure 1f). Similar results were obtained when we analyzed osteogenic differentiation in the different groups, as shown by the lower amount of calcium deposits stained with Alizarin Red (Figure 1g) and a trend for lower expression of osteogenic markers (*ALP, COL1A1, SPP1,* and *RUNX2*) (Figure 1h). By contrast, no differences were found in chondrogenic differentiation between the different hASC groups, as measured by Alcian Blue 8GX staining (Figure 1i) and gene expression of chondrogenic markers (*COMP* and *COL1A1*) (Figure 1j). With the caveat that obesity might bolster some aspects associated with aging, our results suggest that obesity and aging differentially influence hASC properties.

### 2.2 Obesity and aging dysregulate glucose metabolism in hASCs

**FIGURE 2:**
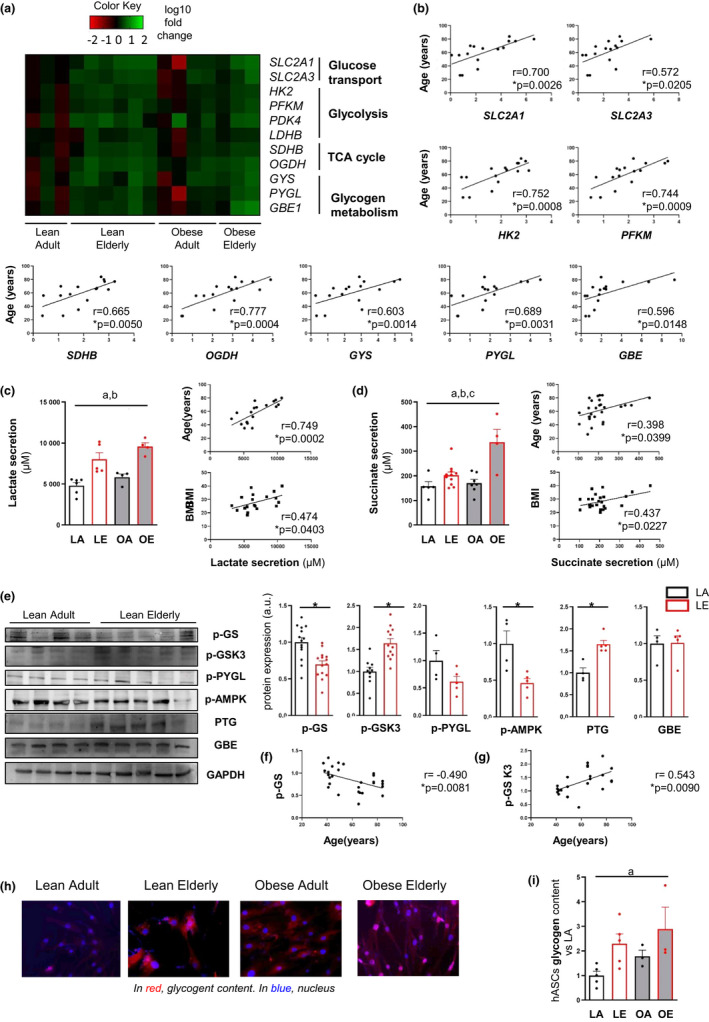
Obesity, but particularly aging, promote glucose utilization by glycolytic and glycogenesis pathways. (a) Gene expression heatmap of glucose transporters (*SLC2A1 and SLC2A3*), glycolytic markers (*HK2, PFKM, and PDK4*), tricarboxylic acid cycle (TCA) enzymes (*LDHB, SDHB, and OGDH*) and glycogen synthesis and degradation enzymes (*GYS, PYGL,* and *GBE1*) (*n* = 3–6). (b) Positive correlation between age and expression of *SLC2A1, SLC2A3, HK2, PFKM, SDHB, OGDH, GYS, PYGL,* and *GBE1* determined by Pearson's correlation analysis. (c) Lactate secretion of hASCs cultured for 24 h and correlation analysis with age and body mass index (BMI) (*n* = 4–6). (d) Succinate secretion of hASCs cultured for 24 h and correlation analysis with age and BMI (*n* = 4–11). Data are shown as mean ± SEM; results of two‐way ANOVA were age *p* < 0.05 (a), BMI *p* < 0.05 (b), interaction between age and BMI *p* < 0.05 (c). Correlations were determined by Pearson's correlation analysis. (e) Representative immunoblots and densitometry of p‐GS, p‐GSK3, p‐PYGL, p‐AMPK, PTG, and GBE protein expression in hASCs from adult and elderly lean donors (*n* = 4–5). (f) Correlation between p‐GS protein expression and age. (g) Correlation between p‐GSK3 protein expression and age. GAPDH was used as a loading control. Densitometry analyses are presented in arbitrary units (a.u). Data are shown as mean ± SEM from three independent experiments; two‐tailed unpaired Student's *t* test, *p* < 0.05 (*). Correlations were determined by Pearson's correlation analysis. (h,i) Glycogen content was determined by indirect immunofluorescence (h) and by a quantitative colorimetric assay (i) (*n* = 3–5). Data are shown as mean ± SEM; results of two‐way ANOVA were age *p* < 0.05 (a), BMI *p* < 0.05 (b), interaction between age and BMI *p* < 0.05 (c). Abbreviations: LA, lean adult; LE, lean elderly; OA, obese adult; OE, obese elderly

We used RNA expression profiling of hASCs to study the impact of obesity and aging on metabolic‐related genes. We found that the expression of several glucose metabolism‐related genes was higher in hASCs from elderly subjects than in adult‐derived hASCs, and this was augmented by obesity (Figure 2a). We also found a significant positive correlation between age and markers of glucose transport (*SLC2A1 and SLC2A3*) and metabolism (*HK2 and PFKM*), tricarboxylic acid cycle (*SDHB, OGDH*), and glycogen metabolism (*GYS, PYGL, and GBE1*) (Figure 2b). The glycolytic phenotype was more pronounced in hASCs from elderly subjects (and was amplified by obesity) and was characterized by a significantly higher secretion of lactate and succinate, which are markers of aerobic glycolysis (Garcia‐Alvarez et al., 2014) and mitochondrial stress (Weinberg et al., 2000), respectively (Figure 2c,d). Lactate and succinate release by hASCs positively correlated with age and BMI (Figure 2c,d). Although we found that both obesity and aging promoted a glycolytic phenotype, the specific upregulation of glycogenic enzymes (*GYS, PYGL,* and *GBE1*), which are known to be expressed in adipocytes (Ceperuelo‐Mallafré et al., 2016), was evident in the groups of elderly subjects (LE and OE) (Figure 2a). As glycogen synthesis is mostly regulated at the protein level, we used immunoblotting to examine different proteins regulating this pathway. Glycogen synthase (GS), the rate‐limiting enzyme in glycogen synthesis, exists in an active (dephosphorylated) and an inactive (phosphorylated) form. The LE group showed significantly lower phosphorylated [p]‐GS levels (inactive GS), which mirrors a higher activity, than the LA group (Figure 2e). Results also revealed significantly higher p‐GSK3 (inactive form of GSK3) levels in hASCs isolated from the LE group (Figure 2e), which agrees well with the activated GS. Moreover, hASCs isolated from the LE group showed higher levels of the glycogen targeting subunit protein targeting to glycogen (PTG). No differences were found in the protein levels of the active form of glycogen phosphorylase (p‐PYGL), which metabolizes glycogen, or in glycogen branching enzyme (GBE), which mediates glycogen branching (Figure 2e). Moreover, the expression of p‐AMPK, which has also been inversely related to glycogen levels (Bijland et al., 2013), was found to be lower in hASCs from the LE group. Correlation analysis showed that p‐GS and p‐GSK3 protein abundance correlated negatively and positively, respectively, with age (Figure 2f,g), suggesting that aging might promote glycogenesis in hASCs. This finding was supported by the evident increase in glycogen content in hASCs from both obese and elderly subjects, as measured by indirect immunofluorescence (Figure 2h). A quantitative colorimetric assay of glycogen (Figure 2i) indicated that age was the main factor influencing glycogen content. Overall, our data reveal that both obesity and aging enhance glucose utilization in hASCs by glycolytic and glycogenesis pathways.

### 2.3 Obesity exacerbates the inflammatory phenotype of hASCs from elderly subjects

**FIGURE 3:**
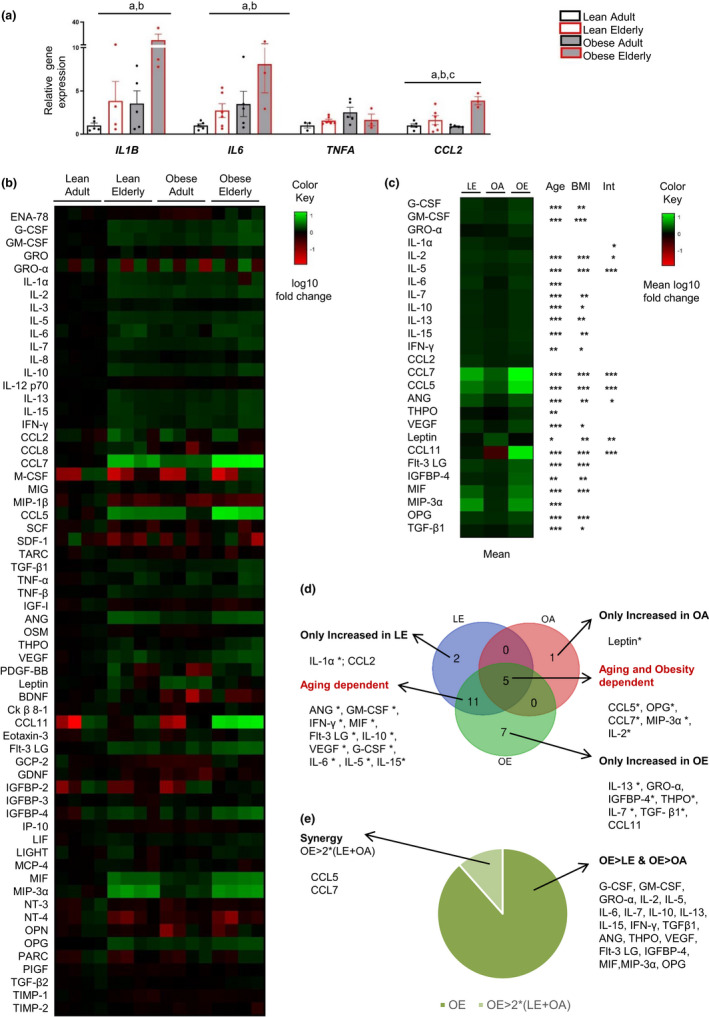
Changes in the cytokine secretion phenotype in hASCs from elderly donors are exaggerated by obesity. (a) Relative gene expression of inflammatory markers (*IL1B, IL6, TNFA,* and *CCL2*) in hASCs from all groups (*n* = 3–6). Data are shown as mean ± SEM; results of two‐way ANOVA were age *p* < 0.05 (a), BMI *p* < 0.05 (b), interaction between age and BMI *p* < 0.05 (c). (b) Heatmap of the 62 cytokines secreted by hASCs of all four groups, expressed as the log10 of the fold change versus hASCs of the lean adult (LA) group (*n* = 5–10). (c) Heatmap of the 26 cytokines secreted by hASCs of the lean elderly (LE), the obese adult (OA) and/or the obese elderly (OE) groups showing ≥1.5‐fold greater levels than those of the LA group, expressed as the log10 of the mean fold change versus the LA group. Results of two‐way ANOVA were age *p* < 0.05, BMI *p* < 0.05, interaction between age and BMI *p* < 0.05. (d) Venn diagram of the 26 cytokines shown in c. (e) Of the 23 cytokines secreted by hASCs from the OE group with ≥1.5‐fold greater levels that those of the LA group, synergy was established for those in this group that showed double the sum of the increase in LE and OA versus LA groups. Abbreviations: LA, lean adult; LE, lean elderly; OA, obese adult; OE, obese elderly

Metabolic reprogramming toward glycolysis is known to be associated with inflammatory states (Soto‐Heredero et al., 2020). In this context, we recently reported a link between glycogen accumulation and pro‐inflammatory cytokine expression in human AT (Ceperuelo‐Mallafré et al., 2016). In line with these studies and with our previous finding of an inflammatory phenotype in hASCs from obese donors (Serena et al., 2016), we noted that the expression of the pro‐inflammatory markers *IL1B, IL6,* and *CCL2* was higher in hASCs from obese adults than from lean adults (Figure 3a). Likewise, age was found to be a major determinant for the expression of the three cytokines. Moreover, a synergistic effect of age and BMI was found for the gene expression of *IL1B* and *IL6*, achieving statistical significance for *CCL2* (Figure 3a). To better understand the effect of aging and obesity on the inflammatory phenotype of hASCs, we used a multiplexed cytokine array (Figure S1) to interrogate the hASC secretome (including chemokines, inflammatory cytokines, and angiogenesis‐ and senescence‐related factors). As shown in Figure 3b, the cytokine secretion pattern of the conditioned medium (CM) of hASCs differed among groups, with the most pronounced changes found in hASCs of elderly subjects (both lean and obese). From the 62 cytokines measured, we focused on those 26 cytokines with a significant increase in ≥1.5‐fold change over hASCs from the LA group in at least one of the groups (Figure 3c). A Venn diagram of the 26 cytokines (Figure 3d) showed that the increase in the abundance of inflammatory cytokines in the hASC‐CM of the OE group was due to aging rather than to obesity, leading us to conclude that aging has a greater influence than obesity on the establishment of a pro‐inflammatory secretome. Nonetheless, a group of five cytokines required the presence of both conditions (aging and obesity) for a significant increase in secretion. Of note, leptin––a classic adipokine––was exclusively increased in the hASC‐CM of the OA group, suggesting that aging counteracts the impact of obesity on leptin secretion. This fits well with previous data describing a negative correlation between leptin plasma levels and age in subjects with obesity (Isidori et al., 2000). Conversely, the abundance of the chemoattractant CCL11 in hASC‐CM was lower in a background of obesity only, and increased markedly in the hASC‐CM of the OE group (Figure 3b,c,d). Indeed, a synergistic induction of chemokine secretion as a response to pro‐inflammatory cytokines has been previously described (Gouwy et al., 2005). To identify whether the presence of both conditions (aging and obesity) could trigger the synergistic induction of some chemokines, we assessed the 23 cytokines secreted by the OE group with a significant ≥1.5‐fold change increase for those with double the sum of the increase in the LE and OA groups. Three chemokines met this criterion: CCL5, CCL7, and CCL11 (Figure 3e). Overall, our data demonstrate that obesity and aging differentially impact the secretory properties of hASCs.

### 2.4 The aged‐ and obesity‐related inflammatory states of hASCs are associated with an antagonistic cross‐talk between glycogen deposition and SIRTs

**FIGURE 4:**
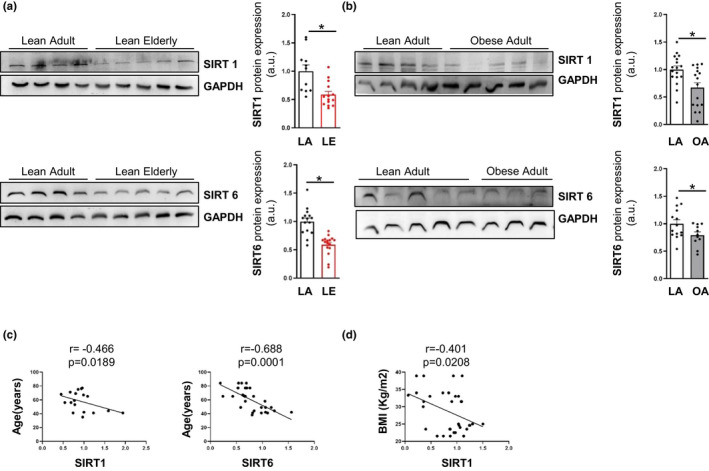
Age‐ and obesity‐related inflammatory status of hASCs negatively associates with SIRT1 and SIRT6 expression. (a,b) Representative immunoblots and densitometry analysis of SIRT1 and SIRT6 protein expression in hASCs of lean adult (LA) and lean elderly (LE) (a) and LA and obese adult (OA) (b) groups (*n* = 3–5). Data are shown as mean ± SEM from three independent experiments; two‐tailed unpaired Student's t test, *p* < 0.05 (*). (c,d) Correlation analysis between SIRT1 or SIRT6 expression and age (c) and between SIRT1 and body mass index (BMI) (d). Correlations were determined by Pearson's correlation analysis. Abbreviations: LA, lean adult; LE, lean elderly; OA, obese adult; OE, obese elderly

Sirtuins are key metabolic sensors involved in the pathophysiology of inflammatory‐related processes including aging and obesity (Vachharajani et al., 2016), and both obesity (Song et al., 2013) and aging (Khanh et al., 2018) have been associated with a reduction in SIRT1 activity in AT. Similarly, published data point to a reduction in SIRT6 protein levels in the AT of people with obesity (Kuang et al., 2017). Thus, we analyzed the expression of SIRT1 and SIRT6 in hASCs from the different groups of donors, finding a downregulation of both in the elderly (Figure 4a) and in the obese (Figure 4b) groups, without significant differences at the mRNA level (data not shown). Correlation analysis showed that both SIRT1 and SIRT6 expression in hASCs correlated negatively with age (Figure 4c), whereas only SIRT1 correlated negatively with BMI (Figure 4d).

**FIGURE 5:**
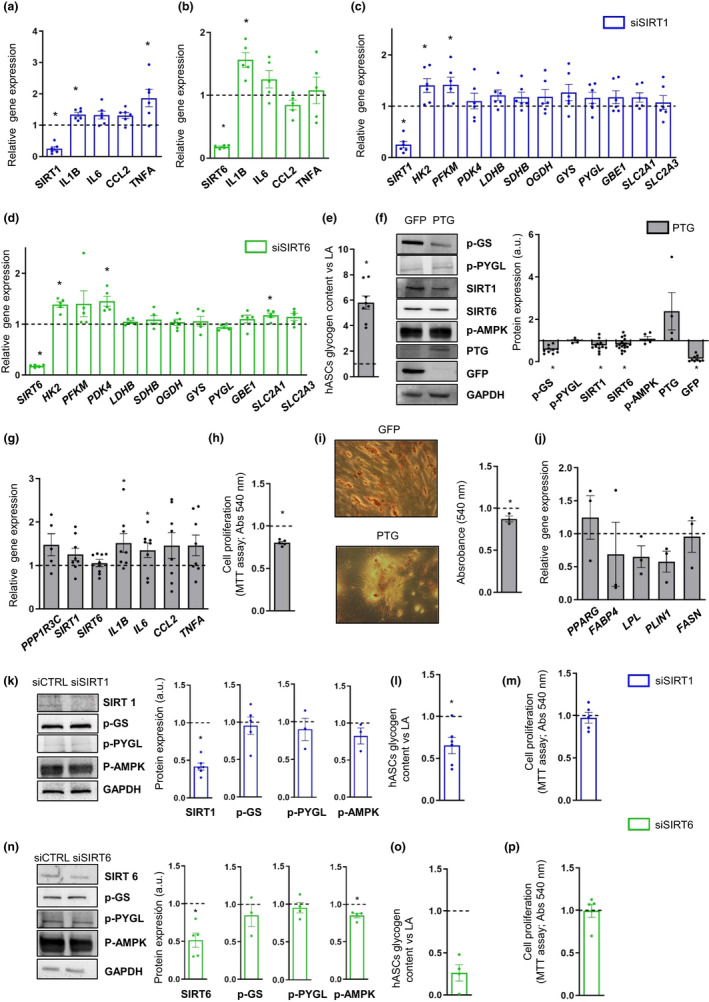
SIRT1 and SIRT6 silencing triggers inflammation and enhances glycolysis in hASCs, whereas enhancing glycogen deposition reduces SIRT1 and SIRT6 protein expression. (a,b) Relative gene expression of *SIRT1* (a) and *SIRT6* (b) and inflammatory markers (*IL1B, IL6, CCL2,* and *TNFA*) in lean adult (LA) hASCs silenced with siSIRT1 (a) (*n* = 6) or siSIRT6 (b) (*n* = 5) versus control hASCs. c,d) Relative gene expression of *SIRT1* (c) or *SIRT6* (d) and glycolytic markers (*HK2, PFKM, and PDK4*), tricarboxylic acid cycle enzymes (*LDHB, SDHB, and OGDH*), glycogen synthesis and degradation enzymes (*GYS, PYGL,* and *GBE1*) and glucose transporters (*SLC2A1 and SLC2A3*) in siSIRT1 (c) (*n* = 6) or siSIRT6 (d) (*n* = 5) hASCs versus control hASCs. (e–g) protein targeting to glycogen (PTG) was overexpressed in hASCs using an adenoviral transduction system. (e,f) LA hASCs overexpressing PTG versus those overexpressing GFP (control) were analyzed for glycogen deposition (e) (*n* = 8) and p‐GS, p‐PYGL, SIRT1, SIRT6, p‐AMPK, PTG, and GFP protein expression (f) (*n* = 3–16). (g) Gene expression of *PPP1R3C, SIRT1, SIRT6* and inflammatory markers (*IL1B, IL6, CCL2,* and *TNFA*) was determined in hASCs overexpressing PTG versus those overexpressing GFP (control) (*n* = 5–8). (h–j) Proliferation determined by MTT assay (h) (*n* = 5) and adipocyte differentiation analyzed by Oil Red O staining (i) (*n* = 3) and gene expression of adipocyte differentiation markers (j) (*n* = 3) were assessed in hASCs upon PTG overexpression versus those overexpressing GFP (control). (k–m) Representative immunoblots and densitometric analysis of SIRT1, p‐GS, p‐PYGL and p‐AMPK (k) (*n* = 3–6), glycogen content (l) (*n* = 6) and proliferation (m) (*n* = 5) in hASCs transfected with siSIRT1 versus control hASCs. (n–p) Representative immunoblots and densitometric analysis of SIRT6, p‐GS, p‐PYGL and p‐AMPK (n) (*n* = 3–5), glycogen content (o) (*n* = 4) and proliferation (p) (*n* = 5) in hASCs transfected with siSIRT6 versus control hASCs. For immunoblots, GAPDH was used as the loading control. Densitometric analyses are presented in arbitray units (a.u.). Data are shown as mean ± SEM; Two‐tailed paired Student's t test, *p* < 0.05 (*)

To test for a link between the decline in SIRT1 and SIRT6 expression and the acquisition of a pro‐inflammatory profile in elderly derived cells, we used short interfering RNA (siRNA) to independently knockdown the expression of *SIRT1* and *SIRT6* in control (LA‐derived) hASCs. Gene expression analysis of inflammatory markers showed that both *SIRT1* (Figure 5a) and *SIRT6* (Figure 5b) knockdown resulted in the upregulation of several pro‐inflammatory genes, including a significant upregulation of *IL1B*. In addition, *SIRT1* downregulation resulted in a significant increase in *TNFalpha* expression (Figure 5a). The inflammatory phenotype of control hASCs induced by *SIRT1* knockdown was accompanied by a significant increase in *HK2* and *PFKM* expression (Figure 5c), whereas *SIRT6* knockdown in control hASCs resulted in a significant increase in *HK2*, *PDK4,* and *SLC2A1* expression (Figure 5d).

Searching for a molecular mechanism underlying SIRT downregulation associated with obesity and aging, we explored metabolic reprogramming as a potential cause. Specifically, we forced glycogen deposition in control hASCs using an adenoviral transduction system overexpressing PTG (Newgard et al., 2000) (Ceperuelo‐Mallafré et al., 2016). As expected, glycogen levels were markedly higher in Ad‐PTG‐hASCs than in control Ad‐GFP‐hASCs overexpressing GFP (Figure 5e), which was consistent with a decrease in p‐GS expression (Figure 5f). We also observed an increase in pro‐inflammatory gene marker expression (*IL1B, IL6, CCL2,* and *TNFalpha*) in PTG‐overexpressing cells, which was significant for *IL1B* and *IL6* expression (Figure 5g), supporting a link between glycogen and inflammation (Ceperuelo‐Mallafré et al., 2016). Notably, SIRT1 and SIRT6 expression was significantly lower in Ad‐PTG‐hASCs than in control cells (Figure 5f), indicating that the downregulation of SIRT1 and SIRT6 in hASCs from elderly and obese donors could be, at least partly, a consequence of glycogen deposition. Contrastingly, no differences in protein expression were found for p‐AMPK (Figure 5f), ruling it out as the upstream interface for SIRT1 and SIRT6 downregulation, at least in these cells. To explore the functional impact of glycogen deposition on hASC function, we assessed cell proliferation and adipocyte differentiation. hASCs overexpressing PTG showed a significant decrease both in proliferation, as determined by MTT reduction (Figure 5h) and in adipocyte differentiation capacity, as revealed by quantitative Oil Red O staining of neutral lipids (Figure 5i), and by gene expression of the adipogenic markers *FABP4*, *LPL,* and *PLIN1* (Figure 5j). Finally, we measured glycogen accumulation in cells silenced for *SIRT1/6* to explore a potential feedback loop between SIRT1/6 and glycogen metabolism. Both *SIRT1* and *SIRT6* knockdown led to a decrease in glycogen content in hASCs without any significant changes to the inactive form of GS (p‐GS) or to the active form of PYGL (p‐PYGL) (Figure 5k,l,n,o), indicating that metabolic dysregulation and glycogen accumulation precedes SIRT1/6 downregulation in aged cells. Moreover, a trend for a decrease in p‐AMPK protein expression was found upon *SIRT1* silencing (Figure 5k), and a significant decrease in p‐AMPK levels was found upon *SIRT6* downregulation, (Figure 5n). These data suggest that p‐AMPK could be downstream of SIRT1 and SIRT6 and linked to the increase of inflammation in response to *SIRT1/6* downregulation. Finally, no differences in proliferation were found in hASCs silenced for *SIRT1* (Figure 5m) or *SIRT6* (Figure 5p). Overall, our data point to a glycogen‐SIRT1/6 axis as a putative driver of age‐related inflammation in hASCs.

## 3 DISCUSSION

Parallels have been drawn between obesity and aging with respect to AT (Pérez et al., 2016), a major contributor to the low‐grade systemic inflammation characteristic of these conditions. Although representing only a fraction of the cells that comprise the AT, adipocyte precursors from the stromal vascular fraction are now regarded as a major source of cytokines and chemokines (Siklova‐Vitkova et al., 2012). Our study provides new insight into the mechanisms governing the dysfunctioning of adipocyte precursors in response to aging and obesity, which activate a metabolic switch to glycogen synthesis that appears to be related to a pro‐inflammatory secretory profile, as we previously reported in adipocytes (Ceperuelo‐Mallafré et al., 2016). In the same line, glycogen metabolism has been recently described as an important event in macrophage‐mediated inflammatory responses (Ma et al., 2020) and in fibroblast‐like synoviocyte‐mediated inflammation (Shi et al., 2018). Our data not only confirm the involvement of glycogen as a driver of inflammation, but also identify SIRT1 and SIRT6 as mediators of this pathogenic event.

While visceral AT (VAT) is known to actively contribute to obesity‐related inflammation and to the development of metabolic disorders, there is emerging evidence for a disruption of both VAT and subcutaneous AT (SAT) homeostasis in an obesogenic context (Lesna et al., 2017). Moreover, recent studies point to a strong correlation between pro‐inflammatory macrophages in SAT and systemic metabolic effects such as insulin resistance (Lesna et al., 2017), supporting the hypothesis that SAT and not VAT status better reflects the systemic state of the subject. Some authors have also reported higher macrophage infiltration in SAT depots of lean subjects with metabolic syndrome when compared to lean healthy individuals, with nearly no differences found in VAT (Moreno‐Indias et al., 2016). These findings could, in part, explain why some normoweight subjects are metabolically unhealthy and point to the SAT depot as the primary detector of metabolic changes (Moreno‐Indias et al., 2016). This is consistent with studies showing that weight loss helps to reduce inflammation particularly in SAT (ClÉment et al., 2004). It is, therefore, conceivable that SAT status better reflects systemic metabolic health. Further work will be needed to determine whether the results obtained in the present study can be extrapolated to hASCs from VAT depots.

The characterization of hASCs modified by aging or obesity revealed that aging could nullify the proliferative advantage of hASCs conferred by an obesogenic background. By contrast, both obesity and aging clearly compromised the differentiation potential of hASCs. These findings are in accord with previous studies from our laboratory (Pachón‐Peña et al., 2016), and with the disrupted differentiation potential reported in senescent mesenchymal stromal cells (Turinetto et al., 2016). Concomitant with these functional alterations, both aging and obesity boosted the overall pro‐inflammatory status of hASCs, but with different patterns. Assessment of the hASC secretome revealed that aging had a more significant impact than obesity, which in turn might boost the aging phenotype. It is known that various types of stimuli trigger changes in the AT secretory pattern in obese and aged individuals toward a more pro‐inflammatory phenotype, which in the case of aging is commonly referred to as the senescence‐associated secretory phenotype (SASP) (Tominaga, 2015). Of note, we also observed an increase in anti‐inflammatory cytokines (e.g., IL‐10 and TGF‐β1) in the CM of hASCs modified by aging or obesity, which fits with the finding that age‐related inflammation is linked to the presence of some anti‐inflammatory factors in the hASC niche (Zhang et al., 2021).

hASCs modified by aging or obesity shared several metabolic hallmarks. As previously described in an obesogenic context (Serena et al., 2016), hASCs from elderly subjects display a glycolytic phenotype, which is similar to that reported in human fibroblasts (Wiley & Campisi, 2016) and hematopoietic stem cells (Poisa‐Beiro et al., 2020) from elderly individuals. Indeed, many studies have described a clear link between glycolysis and inflammatory response (reviewed in (Soto‐Heredero et al., 2020)). We demonstrate here a causal relationship between glycogen deposition and inflammation in adipocyte precursors, extending our previous findings in adipocytes (Ceperuelo‐Mallafré et al., 2016), and showing that hASCs with forced glycogen deposition by an adenovirus overexpressing PTG (Newgard et al., 2000) have a significantly elevated inflammatory response. Abnormal glycogen storage (beyond liver and skeletal muscle) has been described in neurodegenerative diseases (Lafora disease) (Nitschke et al., 2018) and in inflammatory‐related pathological conditions such as obesity (Ceperuelo‐Mallafré et al., 2016), diabetic retinophathy (Gardiner et al., 2015), and rheumatoid arthritis (Shi et al., 2018). We show that glycogen deposition in hASCs reduces their capacity to proliferate and differentiate, indicating that glycogen mishandling alters hASC function.

Consistent with our results in hASCs modified by aging, enhanced glycogen levels have been described in senescent liver (Seo et al., 2008) and in hematopoietic stem cells in an aging context (Poisa‐Beiro et al., 2020). We found that dysfunctional hASCs from elderly and obese individuals have enhanced glucose conversion to glycogen and an aberrant use of glycolytic pathways, two features that seem to be related.

Metabolic dysregulation has long been associated with a reduction in SIRT/NAD activity (Chalkiadaki & Guarente, 2012), and sirtuins have been postulated as sentinels of tissue homeostasis and suppressors of inflammation (Vachharajani et al., 2016). In agreement with previous data showing no significant differences in *SIRT1* and *SIRT6* expression in hASCs from subcutaneous AT depots of individuals with obesity (Mariani et al., 2016), we found no changes across the hASC groups; however, we found a significant decrease in SIRT1 and SIRT6 protein expression in the elderly and obese groups, underlining the important role of post‐transcriptional and post‐translational regulation of sirtuins (Houtkooper et al., 2012). Low protein levels of SIRT1 have been previously reported in the AT of obese mice (Chalkiadaki & Guarente, 2012), and low SIRT6 protein levels were reported in the AT of obese patients (Kuang et al., 2017). Similarly, a reduction in SIRT1 protein expression was found in the AT of aged mice (Gong et al., 2014). Our data support a link between SIRT1/6 downregulation and inflammation in adipocyte precursors, as has been previously demonstrated for macrophages (Yoshizaki et al., 2010). Likewise, it is known that a decrease in the expression of SIRT1 (Gillum et al., 2011) and SIRT6 (Kuang et al., 2017) in AT leads to inflammation. Concomitant with the decline in SIRT1 and SIRT6 levels was a downregulation of p‐AMPK in the elderly group. In addition, a decline in SIRT1/6 translated into a reduction in p‐AMPK protein levels, known to inhibit inflammation in AT (Bijland et al., 2013) and to be responsible for the restoration of glycogen after fasting (Cantó et al., 2010). Therefore, we hypothesize that a decrease in p‐AMPK might be responsible for the enhanced inflammation and the impaired recovery of glycogen levels that occurs following *SIRT1*/*SIRT6* silencing. Given that the inter‐play between glycogen and AMPK is bidirectional, we explored p‐AMPK protein expression in hASCs isolated from the LE group, finding a reduction in its levels. However, no significant differences in p‐AMPK expression were found between hASCs overexpressing PTG and control Ad‐GFP hASCs, suggesting that glycogen accumulation is not the main driver of AMPK inactivation in this context, although this has been described by us in adipocytes (Ceperuelo‐Mallafré et al., 2016), and by others in fibroblast‐like synoviocytes (Shi et al., 2018). These results points to AMPK as a downstream target of SIRT proteins, at least in hASCs.

It is now known that hASCs have important immunomodulatory properties beyond their potential to differentiate into adipocytes. Analogous to other AT immune cells, hASCs might also control tissue remodeling in response to specific challenges such as overnutrition and aging. We and others have demonstrated that obesity disturbs this dual function of hASCs (Serena et al., 2016), which is reflected in both a local pro‐inflammatory phenotype and in the inability to properly store triglycerides in AT. We propose that similar to what occurs in obesity, the hostile environment associated with aging induces significant changes in hASCs primarily to respond to the inflammation in the tissue, at the expense of differentiation potential. Therefore, the reduced proliferation and differentiation of “elderly” hASCs impairs the expansion of SAT necessary to adequately respond to energy excess. The expansion of AT through differentiation of hASCs into new adipocytes (termed hyperplasia) is a counteracting mechanism to prevent lipids being stored in other organs, in response to chronic positive energy balance. Elderly subjects commonly present with elevated levels of serum‐free fatty acids, dyslipidemia, insulin resistance (Pararasa et al., 2015) and a redistribution of fat toward ectopic depots, which are likely consequences of a dysfunctional AT with hASCs with reduced adipocyte differentiation capacity.

Overall, our study reveals a novel pathway linking metabolic dysfunction to inflammation in adipocyte precursors, particularly in the context of aging and obesity (figure 6, graphical abstract). While glycogen accumulation has previously been described as a driver of inflammation in other cells and pathologies, this is the first time that SIRT1 and SIRT6 have been reported as mediators between the two processes. Perpetuation of inflammation in AT would be aggravated by a metabolic switch in hASCs to aerobic glycolysis, which enhances glycogen deposition. Our work points to sirtuins as possible mediators linking both features. Further studies in vivo will be required to determine whether switching back to fatty acid oxidation, reducing glycogen levels or restoring SIRT1/6 levels in hASCs might prevent local and systemic inflammation.

## 4 EXPERIMENTAL PROCEDURES

### 4.1 Study subjects

Subjects were recruited at the University Hospital Joan XXIII and at Santa Tecla Hospital in accordance with the tenets of the Helsinki Declaration. The corresponding hospital ethics committees reviewed and approved the study and written informed consent was obtained from all participants before entering the study. SAT biopsies were obtained from donors undergoing nonacute surgical interventions, such as hernia or cholecystectomy, in a scheduled routine surgery. Donors were classified as adult (>20 and < 65 years) or elderly (≥65 years) based on their age; and as lean (BMI < 25 kg/m^2^) or obese (BMI≥30 kg/m^2^) based on their BMI, following World Health Organization criteria. This classification led to formation of four groups lean adult (LA), lean elderly (LE), obese adult (OA), and obese elderly (OE). The anthropometric and biochemical variables from the cohort are presented in Table S1. Patients with cancer, diabetes and inflammatory chronic diseases were excluded from the study. The *n* corresponding to the subsample used for each experiment is specified in the figure legends.

### 4.2 Isolation and culture of human adipose‐derived mesenchymal stromal cells

hASCs were isolated from SAT biopsies as described (Serena et al., 2016; Pachón‐Peña et al., 2016). In brief, SAT was washed extensively with PBS to remove debris and treated with 0.1% collagenase in PBS and 1% bovine serum albumin for 1 h at 37°C with gentle agitation. Digested samples were centrifuged at 300 × *g* at 4°C for 5 min to separate adipocytes from stromal cells. The cell pellet containing the stromal fraction was resuspended in stromal culture medium consisting of DMEM/F12, 10% fetal bovine serum (FBS), and 1% antibiotic/antimycotic solution and then placed into a flask. The flask was placed in an incubator at 37°C with 5% CO_2_ and 21% O_2_. At 24 h after seeding, the flask was washed with PBS and the medium was replaced. As the hASCs had already adhered to the flask, the PBS wash removed only non‐adherent cells. Culture medium was replaced every 48–72 h. After 7 days of incubation, when cells had achieved 90% confluence, the primary cultures of hASCs at P0 were harvested with trypsin–EDTA, and aliquots of 1 × 10^6^ cells were cryopreserved in liquid nitrogen until required. The AT‐cell number ratio was defined as the ratio of the number of cells obtained at P0 per gram of AT digested. CM was collected at P3–7 after 24 h in culture using a minimum concentration of hASCs of 10,000 cells/cm^2^ and was centrifuged at 400 × *g* for 5 min.

### 4.3 Immunophenotyping

To verify the isolation of hASCs, we assessed the immunophenotypic profile of undifferentiated hASC populations using a panel of positive and negative surface markers that identify hASCs according to the quantitative criteria established by the International Society of Cell Therapy (ISCT) and the International Federation for Adipose Therapeutics and Science (IFATS). Briefly, 2 × 10^5^ hASCs were incubated with a panel of primary antibodies (CD34, CD73, CD90, CD105, CD14, CD31, and CD45) and then analyzed by flow cytometry using 405‐nm, 488‐nm, and 633‐nm excitation on the FACS ARIA III cytometer (BD Biosciences, San Jose, CA). All experiments were performed in cells at P3–7. Flow cytometry analysis of cell marker expression was consistent with the minimum criteria defined for hASCs. Accordingly, cells were positive for the surface markers CD73, CD90, and CD105 and negative for CD34, CD14, CD31, and CD45. No significant differences were detected between groups (Table S2).

### 4.4 Proliferation assays

#### 4.4.1 MTT assay

hASC proliferation was determined by a standard colorimetric 3‐(4,5‐dimethylthiazol‐2‐yl)‐2,5‐diphenyltetrazoliumbromide (MTT) reduction assay (Sigma‐Aldrich, Madrid, Spain). In brief, 1.6 × 10^3^ hASCs per well were seeded in a 96‐well plate. Two MTT assays were performed at 24 h (day+1) and 7 days (day+7) after seeding. The proliferation rate was calculated as the difference in absorbance between day+7 and day+1, measured with a spectrophotometer at 540 nm.

#### 4.4.2 CTV assay

Flow cytometric analysis of intracellular CTV (Invitrogen, Eugene, OR) was used to measure hASC proliferation, based on dye dilution of CTV‐labeled cells over the course of 48 h.

### 4.5 Multilineage differentiation capacity

To assess the differentiation capacity of hASCs, we used specific conditions to trigger cell differentiation to the adipocyte, osteocyte, and chondrocyte lineage, as described (Bunnell et al., 2008). Intracellular lipid enrichment in mature adipocytes was measured by Oil Red O staining; calcium depots in osteocytes were assessed by Alizarin Red staining; and glycosaminoglycan precipitation in chondrocytes was analyzed by Alcian Blue 8GX staining. Differentiated cells were observed in a bright‐field microscope (Zeiss Axio Vert A1; Carl Zeiss AG, Oberkochen, Germany). Relative gene expression of adipogenic osteogenic and chondrogenic markers in hASCs was analyzed by real‐time polymerase chain reaction (Figure S2).

### 4.6 Cytokine secretion

We used pools of 24 h CM from hASCs of different donors (at least an *n* = 5 was used for each group), of which 500 μL samples were filtered using a 3‐k pore filter and centrifugated at 14000 × *g* for 5 min. A total of 100 μl was used in duplicate for each cohort on the RayBio Human Cytokine Antibody Array 5 (G series, cat# AAH‐CYT‐G5‐8, RayBiotech Life; www.raybiotech.com), and the array was sent to the manufacturer for scanning. Fluorescence signal intensities were measured on the Innopsys InnoScan (Carbonne,) laser fluorescence scanner. Normalization was performed by defining one of the two sub‐arrays of the LA group as a reference. The image of the array is shown in supplementary data (Figure S1).

### 4.7 Gene expression analysis

Total RNA was isolated from hASCs using the RNeasy Lipid Tissue Mini Kit (Qiagen Science,). RNA was transcribed to cDNA with random primers using the Reverse Transcription System (Applied Biosystems,). Amplification was performed on a 7900HT Fast Real‐Time PCR System using the TaqManR Gene Expression Assays hydrolysis probes (Applied Biosystems) (Figure S2). Results were calculated using the comparative threshold cycle (Ct) method (2^−ΔΔCt^) normalized to the expression of the housekeeping gene *18S* (Hs 03928985_g1) or cyclophilin 1A (*PPIA*) and expressed relative to the control condition, which was set to 1. Two technical duplicates were performed for each biological replicate.

### 4.8 Protein expression analysis

Cells were lysed and homogenized in Mammalian Protein Extraction Reagent (M‐PER™; ThermoFisher Scientific,) containing a protease and phosphatase inhibitor cocktail (Sigma‐Aldrich). The BCA Protein Assay Kit (Pierce Biotechnology,) was used to determine protein concentration. Equal amounts of protein were separated on SDS‐PAGE gels, transferred to Immobilon membranes (Merck Millipore,) and blocked. Antibodies diluted 1/1000 against p‐AMPK (THR172 [40H9]) (2535; Cell Signaling Technology [CST]), p‐GS (SER641) (3891; CST), p‐GSK3 α/β (SER21/9) (9331; CST), GBE (AB617523; Abcam), GFP (8371–2; Clontech), PTG (SC‐6582; Santa Cruz Biotechnology),

p‐PYGL(S15) (AB227043; Abcam), SIRT1 (2310, CST), SIRT6 (AB88494; Abcam), were used to perform immunoblot analysis. GAPDH (MA5–15738; Sigma‐Aldrich) was used as a loading control. Protein bands were detected with anti‐rabbit (NA934; GE Healthcare,) or anti‐chicken (ab131366; Abcam) peroxidase‐conjugated secondary antibodies, diluted 1/2000. Immunoreactive bands were visualized using a SuperSignal West Femto chemiluminescent substrate (Pierce) and images were captured on a “iBrightCL1000 image System.” ImageJ software (NIH) was used to quantify the intensity of the bands.

### 4.9 Glycogen immunofluorescence

The monoclonal anti‐glycogen antibody used for the immunofluorescence was generously provided by Dr. Otto Baba (Baba, 1993). hASCs grown on coverslips were fixed with 4% (w/v) paraformaldehyde, rehydrated with 2% (v/v) fish skin gelatin, and permeabilized with 0.2% Triton X‐100 prior to incubation with 5% (v/v) goat serum. Subsequently, cells were incubated overnight at 4°C with the monoclonal mouse anti‐glycogen antibody in PBS containing 1% goat serum. Coverslips were washed with PBS and incubated for 1 h at room temperature with an Alexa Fluor 568 conjugated goat anti‐mouse antibody (1:100) and then mounted with ProLong Gold Antifade Reagent containing 40,6‐diamidino‐2‐phenylindole (DAPI) (Invitrogen). Microscopy was performed with a Leica DM 4000B fluorescence microscope (Leica Microsystems), and images were captured with a Leica DFC 300 FX camera (Leica Microsystems).

### 4.10 Glycogen colorimetric assay

Glycogen levels were measured in hASCs using the Glycogen Colorimetric Assay Kit (BioVision Inc.,). In total, 3 × 10^5^ hASCs were homogenized with 200 μl of water on ice, and the assay was performed following manufacturer's instructions. A glucose background control was determined and then subtracted from the glycogen readings.

### 4.11 Adenoviral transduction

Cells were infected 1 day after seeding with an adenovirus expressing murine PTG (Ad‐PTG) or GFP (Ad‐GFP) under the control of the CMV promoter (Gasa et al., 2000). The adenovirus expressing PTG or GFP (used as a control) was diluted in Opti‐MEM Reduced Serum Medium (Gibco, ThermoFisher Scientific) at 1/200 and 1/4000, respectively, prior to use. Adenoviral infection was carried out for 2 h at 37°C using a multiplicity of infection of 50. The medium containing the adenovirus was then removed and replaced with standard culture medium. Two days after infection, culture medium was collected, and hASCs were collected and frozen.

### 4.12 SIRT1 and SIRT6 silencing

Silencing consumables were all from Horizon Discovery. Cells seeded at 10,000 cells/cm^2^ were transfected with human SIRT1 siRNA (003540) or SIRT6 siRNA (013306) or a control (On‐Target Plus Non‐targeting Pool, number 001810). siRNA (5 μM) and Dharmafect Transfection Reagent were diluted 1/20 and 1/25, respectively, in Opti‐MEM Reduced Serum Medium (Gibco; ThermoFisher Scientific) and incubated for 5 min at room temperature. The same amount of each solution was mixed carefully and incubated for 20 min at room temperature. The final solution of siRNA at a concentration of 0.125 μM was added to hASCs and medium containing serum was also added to the wells. hASCs were incubated at 37°C with 5% CO_2_ for 72–96 h.

### 4.13 Statistical analysis

Statistical analysis was performed with GraphPad Prism 8 (GraphPad Software Inc.,). For in vitro data, experimental results were presented as mean ± SEM from 3 to 5 independent donors for each experiment. Statistical significance was tested by parametric two‐way analysis of variance (ANOVA), when 4 groups were analyzed, and by the parametric Student's unpaired t test when two groups were analyzed. For the gain or loss of function studies, the parametric Student's paired *t* test was used. Correlations were tested by Pearson's correlation analysis. General linear model and multiple linear regression analyses were employed to exclude sex as a confusing factor in the differences in proliferation found between groups.

## AUTHOR CONTRIBUTIONS

M.T–P. contributed to conception and design, provision of study material or patients, collection and/or assembly of data, conduction of experiments, data analysis and interpretation, manuscript writing, and final approval of the manuscript. I.H‐R. contributed to conception and design, data analysis and interpretation, and final approval of the manuscript J.S‐B., M.E., E.M‐M., and C.N‐R. conducted the experiments. C.S. contributed to data analysis and interpretation. J.V. contributed to financial support, administrative support, and final approval of the manuscript. S. F‐V. contributed to conception and design, financial support, administrative support, data analysis and interpretation, manuscript writing, and final approval of manuscript.

## CONFLICT OF INTEREST

The authors declare that they have no competing interests.

## CONSENT TO PARTICIPATE

Informed consent was obtained from all subjects involved in the study. Subjects were recruited by the Endocrinology and Surgery departments at the University Hospital Joan XXIII. The cells were stored in a tissue biobank registered at the National Register of Biobanks (registration number #C.0003609).

## Supporting information


Appendix S1

## Data Availability

The datasets used and/or analyzed during the current study are available from the corresponding authors on reasonable request.
