# Supplementary material for: Glycogen accumulation in adipocyte precursors from elderly and obese subjects triggers inflammation via SIRT1/6 signaling
Source: Aging Cell. 2022 Jul 10;21(8):e13667. doi: 10.1111/acel.13667 (PMC9381900; doi:10.1111/acel.13667)
Supplement: Supplementary file 1 — Appendix S1 [file ACEL-21-e13667-s001.pdf]

Figure S.1 Raybio human cytokine antibody array Array

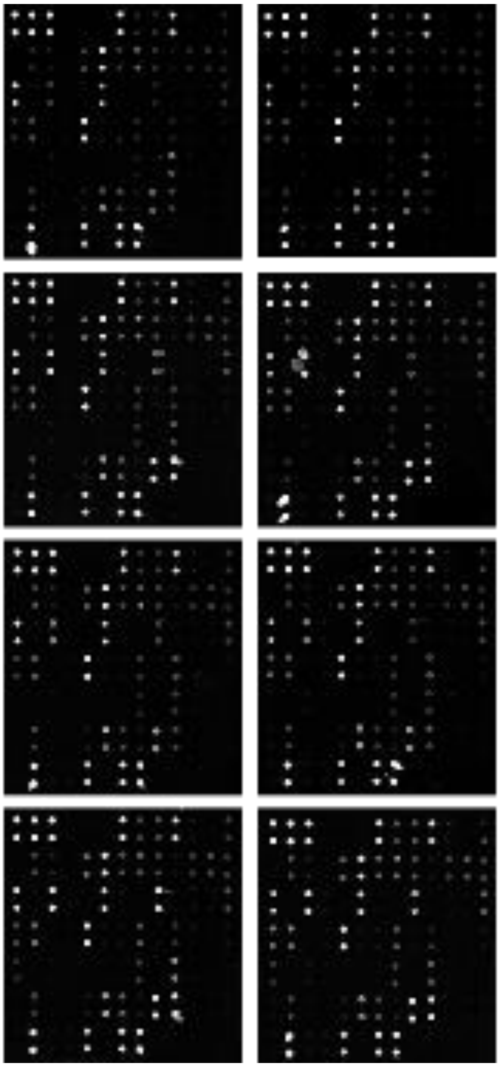

Lean Adult
Sample 1

Lean Adult
Sample 2

Lean Elderly
Sample 3

Lean Elderly
Sample 4

Obese Adult
Sample 5

Obese Adult
Sample 6

Obese Elderly
Sample 7

Obese Elderly
Sample 8

Figure S.2. Human gene expression analysis

Gene symbol-Assay ID	Gene nane
**Senescence markers**	
**CDKN2A**-Hs00923894_m1	Cyclin Dependent Kinase Inhibitor 2A
**GLB1**-Hs01035168_m1	Galactosidase Beta 1
**TP53**-Hs01034249_m1	Tumor Protein P53
**Adypocyte differentiation markers**	
**FABP4**-Hs01086177_m1	Fatty Acid Binding Protein 4
**FASN**-Hs01005622_m1	Fatty Acid Synthase
**LPL**-Hs00173425_m1	Lipoprotein Lipase
**PLIN1**-Hs00160173_m1	Perilipin 1
**PPARG**-Hs01115513_m1	Peroxisome Proliferator Activated Receptor Gamma
**Osteocyte differentiation markers**	
**ALPL**-Hs01029144_m1	Alkaline Phosphatase
**COL1α1**-Hs00164004_m1	Collagen Type I Alpha 1 Chain
**RUNX2**-Hs01047973_m1	RUNX Family Transcription Factor 2
**SPP1**-Hs00959010_m1	Secreted Phosphoprotein 1/Osteopontin
**Condrocyte differentiation markers**	
**COL1α1**-Hs00164004_m1	Collagen Type I Alpha 1 Chain
**COMP**-Hs00164359_m1	Cartilage Oligomeric Matrix Protein
**Metabolism markers**	
**GYS_1**-Hs00157863_m1	Glycogen Synthase 1
**GBE_1**-Hs00609186_m1	1,4-Alpha-Glucan Branching Enzyme 1
**HK2**-Hs00606086_m1	Hexokinase 2
**LDHb**-Hs00929956_m1	Lactate Dehydrogenase B
**OGDH**-Hs01081865_m	Alpha-ketoglutarate dehydrogenase
**PDK4**-Hs01037712_m1	Pyruvate dehydrogenase kinase, isozyme 4
**PFKM**-Hs00175997_m1	Phosphofructokinase M
**PPP1R3C**-Hs00193642	Protein Phosphatase 1 Regulatory Subunit 3C/ Protein Targeting To Glycogen (PTG)
**PYGL**-Hs00958087_m1	Glycogen Phosphorylase L
**SDH_b**-Hs01042482_m1	Succinate dehydrogenase b
**SLC2A1/GLUT1-**Hs00892681_m1	Solute Carrier Family 2 Member 1/Glucose Transporter Type 1
**SLC2A3/GLUT3-**Hs00892681_m1	Solute Carrier Family 2 Member 3/Glucose Transporter Type 3
**Inflammation markers**	
**CCL2**-Hs00234140_m1	C-C Motif Chemokine Ligand 2
**IL1B**-Hs01555410_m1	Interleukin 1 Beta
**IL6**-Hs00174131_m1	Interleukin 6
**TNFA-**Hs00174128_m1	Tumor necrosis factor alpha
**Sirtuins**	
SIRT1-Hs01009005_m1	Sirtuin 1
SIRT6-Hs00213036_m1	Sirtuin 6

Results were calculated using the comparative Ct method and expressed relative to the expression of the housekeeping genes cyclophilin 1A (PPIA) (Hs04194521_s1) and 18S (Hs03928985_g1)

Table S.1. Anthropometric and biochemical variables from the cohorts used to obtain human adipose-derived mesenchymal stromal cells (hASCs)

	Lean Adult	Lean Elderly	Obese Adult	Obese Elderly
**n**	29	16	30	8
**Sex (male/female)**	12/17	10/6	17/13	0/8
**Age (years)**	45.3±9.8^{b,d}	70.9±6.7^{a,c}	48.7±8.5$^{b;d}$	72±4.7^{a,c}
**BMI (kg/m^2)**	23.6±2.7^{c,d}	24.7±2.3^{c,d}	34.3±4.5^{a,b}	33.3±3.5^{a,b}
**Glucose (mmol/L)**	4.86±0.97	4.81±1.13	5.43±0.99	5.82±0.94
**Total Cholesterol (mmol/L)**	4.69±0.93	5.25±1.19	5.14±1.19	5.20±0.59
**HDLc (mmol/L)**	1.46±0.48	1.54±0.34	1.20±0.37	1.45±0.41
**LDLc (mmol/L)**	2.68±0.90	3.26±0.85	3.19±0.98	3.02±0.49
**Triglycerides (mmol/L)**	1.18±0.70^{c}	1.09±0.32^{c}	1.89±0.82^{a,b}	1.60±0.54

Abbreviations

BMI: body mass index; HDLc: high-density lipoprotein cholesterol; LDLc: low-density lipoprotein cholesterol. Results are given as mean±SD. ANOVA followed by post hoc Bonferroni was used to compare means between groups: ^{a} *P* < .05 *vs* Lean Adult; ^{b} *P* <.05 *vs* Lean Elderly, ^{c} P<.05 vs Obese Adult; ^{d} P<:05 vs Obese Elderly

Table S.2. Immunophenotypic profile of undifferentiated human adipose-derived mesenchymal stromal cells (hASCs) isolated from adult and elderly individuals[a]

Surface markers

	**Adult**		**Elderly**	
	%	MFI	%	MFI
**CD34**	0.52±0.52	100±67	0.71±0.63	130±117
**CD73**	92.63±3.15	6187±2985	93.29±5.57	7825±3533
**CD90**	89.42±8.21	11775±8203	89.27±9.19	12022±11674
**CD105**	70.28±17.15	2722±2061	78.69±14.66	2318±1280
**CD14**	0.33±0.28	115±40	0.25±0.27	96±25
**CD31**	0.54±0.37	188±81	0.56±0.58	234±157
**CD45**	0.29±0.21	121±69	0.28±0.37	132±62.10

Human adipose-derived mesenchymal stromal cells obtained from subcutaneous adipose tissue biopsies from Adult and Elderly donors were stained with the panel of antibodies described and analyzed by flow cytometry using 405-nm, 488-nm and 633-nm excitation on the FACSAREA III cytometer (BD). Values are reported as the mean ±SD and means were compared between groups with the Student's unpaired t-test:
[a] $P<.05$ *vs* Lean Adult
Abbreviation: MFI, mean fluorescence intensity (arbitrary units)
